# Sociocultural and Demographic Risk Factors for the Development of Multiple Sclerosis in Kuwait: A Case - Control Study

**DOI:** 10.1371/journal.pone.0132106

**Published:** 2015-07-01

**Authors:** Suhail N. Al-Shammri, Magdy G. Hanna, Arpita Chattopadhyay, Abayomi O. Akanji

**Affiliations:** 1 Neurology Unit, Mubarak Al Kabir Hospital, Ministry of Health, Jabriya, Kuwait; 2 Division of Neurology, Department of Medicine, Faculty of Medicine, Jabriya, Kuwait University, Kuwait; 3 Department of Medical Sciences, Frank H. Netter, MD, School of Medicine, Quinnipiac University, Hamden, Connecticut, United States of America; University of Oxford, UNITED KINGDOM

## Abstract

**Introduction:**

Immunological, genetic and environmental factors are believed to play important roles in the pathogenesis of Multiple Sclerosis (MS). There have been many studies on risk factors for MS but these have been mainly in Caucasian populations; robust studies in Arab populations remain relatively uncommon. This study therefore aimed to identify behavioral, socio-cultural, and demographic factors associated with development of MS in Kuwait, a high income Arab country, currently undergoing a demographic transition.

**Subjects and methods:**

In this case- control study, 195 Kuwaiti MS patients and 146 healthy age and sex-matched controls were recruited. Both groups of subjects were interviewed using a structured questionnaire, in relation to anthropometric, socio-cultural and demographic data, residence during the 1990/91 Gulf War and current and past medical history, including medications. We also clinically evaluated, and retrospectively reviewed medical records of patients to derive appropriate clinical information, including associated chronic medical illness requiring long-term treatment.

**Results:**

On multiple logistic regression analysis after adjustment for potential confounders including age, gender and BMI, in all the subjects, a positive associations prevail with presence of MS and some sociocultural and demographic factors, which included non-Bedouin ethnicity (AOR 2, 95% CI 1.0-3.9, p 0.049), positive family history of MS (AOR 10.6, 95% CI 3.0-36.9), p < 0.001), and low daily sunlight exposure of < 15min/day (AOR 5.3, 95% CI 2.7-10.5 p < 0.001). In addition, while 41.8% of MS patients indicated at least one comorbidity, only 26.8% of the controls reported any associated physical illness, with the suggestion that presence of certain comorbidities might increase MS risk (AOR 2.4, 95% CI 1.3-4.7, p < 0.001). Other risk variables such as smoking status and mode of routine outdoor dressing were not significant in all the MS subjects taken as a whole, but demonstrated variably positive associations in the MS subgroup classified as those with established disease and those who were newly diagnosed and drug naïve.

**Conclusions:**

This study suggests that a positive family history of MS and presence of certain comorbidities appeared to be associated with an increased risk of developing MS. In contrast, relatively increased amount of daily sunlight exposure and Bedouin ethnicity appear to somewhat be protective. It is speculated that the relationship of sunlight exposure with MS might be due to vitamin D availability, and is deserving of further study.

## Introduction

Multiple sclerosis is an inflammatory disease of the central nervous system of presumed autoimmune origin. It is widely believed that both genetic and environmental factors are involved in its etiopathogenesis [1, 2].

Environmental influence on MS susceptibility is suggested by the striking geographical distribution of disease prevalence (including increasing prevalence from increasing latitudinal distance from equator), and incomplete concordance in monozygotic twins [3].

Despite decades of epidemiological, clinical and laboratory research, however the specific etiologic mechanisms for MS remain largely speculative [1].

The risk factors that have been robustly associated with involvement in MS are Epstein-Barr virus (EBV) infection, cigarette smoking, sunlight exposure and vitamin D deficiency. These risk factors and other socio-demographic and lifestyle factors have been rigorously explored in high prevalence Caucasian populations but data from traditionally low prevalence regions such as those in the Middle East have been sparse [2].

Kuwait is a small country, with fairly homogenous ethnicity, situated on the North-east of the Arabian Peninsula at the Northern end of the Arabian Gulf; (latitude 28–30° N). The 2005 population is about 3.27 million, of whom just less than 50% (about 1.13m), are Kuwaiti citizens of Arab descent. Most of the other residents are non-citizens from other countries, especially other Arab countries and the Indian sub-continent. Most Kuwaiti citizens can trace their ancestry to Arab or Persian roots. Historically, the population of Kuwait comprises early settlers originating from the tribes of neighboring Arabian and Persian countries (particularly Saudi Arabia and Iran), and the nomadic Arabs of the desert, living in the fringes of the Arabian Peninsula [4]. There has been a tremendous ethnic shift in the population since the Gulf War in 1990–91, with the departure of the Palestinians who made up the majority of the non-Kuwaiti Arab expatriates prior to the invasion by Iraq. The Gulf War also brought huge numbers of troops into the country from America and Europe.

Multiple sclerosis is now believed to be much more common in Kuwait than earlier presumed—its prevalence has increased from 9.5/100,000 in 1988 to 14.7/100,000 in 2000. Indeed, a recent retrospective study suggested that Kuwait has emerged as a high prevalence area with a crude prevalence of 78.04/100,000 [5–7]. The reasons for this increased prevalence are essentially unclear and deserving of further study. We therefore devised this case—control study of putative environmental and socio-demographic risk factors for MS in groups of Kuwaiti subjects with and without diagnosed MS.

## Subjects and Methods

### Subjects

In this case-control study, we recruited 195 Kuwaiti MS patients from the national MS clinic at Mubarak Al-Kabir Specialist Hospital in Kuwait. All these patients were diagnosed as per the McDonald criteria [8], and followed up by our team of experienced neurologists. We also recruited 146 healthy age and sex matched controls randomly from the Kuwaiti community living in Kuwait.

Each subject was interviewed using a structured questionnaire to collect specific information related to:

1. Anthropometry—height, weight and BMI.
2. Demography—age, gender, nationality, ethnicity and country of refuge during the Gulf War (1990/91).
3. Socio-cultural: history of smoking (current and past), consanguinity, family history of MS, daily amount of sun exposure, preferred choice of routine outdoor dressing (full or partial body cover).

All the subjects were clinically evaluated, and had their available medical records retrospectively reviewed for relevant past medical history, medications and other pertinent clinical information. Such information included evidence for other associated chronic medical illness requiring long-term treatment. Thereafter, those patients with MS were classified according to (i) established disease vs. newly diagnosed drug-naïve disease, and (ii) their clinical phenotypes (relapsing-remitting, secondary progressive and primary progressive) and disease stage using the Expanded Disability Status Scale (EDSS) [9]. In addition, overnight fasting blood specimens were collected for assessment of complete blood counts and renal and liver function, by routine methods.

### Statistical methods

Statistical analyses were performed using the Statistical Package for Social Sciences, SPSS for Windows (IBM SPSS Statistics 22, IBM Corporation, Armonk, NY, USA 2013). The level of statistical significance was set at p < 0.05. The chi-square test or Fisher’s exact test was used to assess the association between two categorical variables wherever appropriate. Quantitative variables were represented as mean± standard deviation (mean ± SD) or median (interquartile range (IQR) as appropriate. Quantitative variables were compared between MS patients and controls using an independent samples t-test or Mann-Whitney U test depending on the sample distribution. The multiple logistic regression analysis was used to estimate the risk of these factors in the prevalence of MS after controlling for confounding between them. The variables age, sex, BMI, ethnicity, time spent in Kuwait during the 1990/91 Gulf War, family history of MS, history of associated diseases, daily sunlight exposure duration and type of outdoor clothing routinely worn, were included in the model to control for confounding. The AOR (95% CI) for the identified associations were reported, as considered appropriate.

### Ethics Statement

This study was approved by the Kuwait University Health Science Centre Ethical Committee. Every subject signed an informed written voluntary consent form.

## Results

Of the total of 195 patients recruited into the study, 61 were newly diagnosed and not taking any disease modifying drugs at time of recruitment and 134 patients had established disease and were on disease modifying drugs. Characteristics of the patient population and their clinical phenotypes are indicated in Table 1. In addition, 81 MS patients and 33 controls took regular nutritional supplements including multivitamins. With respect to clinical disease phenotypes and disease stage, 166 (85.2%) patients had relapsing remitting MS (RRMS); 26 (13.5%) were secondary progressive (SPMS); and 3 (1.5%) had primary progressive (PPMS) disease. The majority [n = 155 (79.5%)] had benign disease as suggested from their median EDSS 1.5 (0–2.5) (Table 1). The patients were mostly young to middle-aged [median (IQR):32 (27–38) yrs] and had been diagnosed with MS while aged < 30 years [median (IQR):28 (21–34) yrs], with the onset of symptoms at about a median (IQR) age of 27 (20–33) years (Table 1).

**Table 1:** Characteristics of the patient population.

Variables	All MS Patients	Established MS patients	Newly diagnosed patients (drugs naïve)
Total number of subjects (N)	195	134	61
**MS subtype:**			
Relapsing Remitting, N (%)	166 (85.2)	105 (78.4)	61 (100)
Secondary Progressive, N (%)	26 (13.3)	26 (19.4)	-
Primary Progressive, N (%)	3 (1.5)	3 (2.2)	-
Age at recruitment (years), median (IQR)	32 (27–38)	33 (27–41)	31 (26–36)
Age at diagnosis (years), median (IQR)	28 (21–34)	27 (20–33)	30.5 (23.8–34.0)
Age at onset (years), median (IQR)	27 (20–32)	26 (19–32)	28.5 (22.0–32.8)
MS duration (years), median (IQR)	3 (0–8)	5 (2–10)	2 (1–7)
EDSS score, median (IQR)	1.5 (0–2.5)	1.5 (0–3)	1 (0–2)
Number of relapses, median (IQR)	3 (1–5)	4 (2–7)	0.1 (0.1–0.2)

Table 2 summarizes the anthropometric, demographic and sociocultural characteristics of both the patients’ and controls’ groups. It is seen that, while the mean ages of both control and patient groups were essentially similar at recruitment, those patients with newly diagnosed disease were significantly younger than controls (30.8±8.3yrs. vs 33.8±9.5 yrs.; p 0.029) (Table 2). There were more females than males, (ratio 1.6:1). The univariate analyses showed that more MS patients than controls were of non-Bedouin ancestry (p 0.016), smoked (p 0.026), stayed in Kuwait during the 1990/91 Gulf War (p 0.017), had a positive family history of MS (p < 0.001), they also had certain other comorbid illnesses (p 0.005). The MS patients also tended to have lesser daily sunlight exposure (p < 0.001) (Table 2). These associations were then further explored by multiple logistic regression analyses with appropriate correction for confounding (see Statistical Methods above).

**Table 2:** Demographic, sociocultural and anthropometric characteristics of MS patients and controls.

	Controls	All MS patients	p *	Established MS Patients	p ^†^	Newly diagnosed patients (drugs naïve)	p ^‡^
Total number of subjects (N)	146	195		134		61	
Variables	N (%)	N (%)		N (%)		N (%)	
**Anthropometric Parameters**							
**BMI (kg/m^2^)**			0.359^a^		0.241^a^		0.933^a^
Normal (<25)	37 (27.2)	64 (34.6)		47 (36.7)		17 (29.8)	
Overweight (25–29.99)	50 (36.8)	59 (31.9)		39 (30.5)		20 (35.1)	
Obese (≥30)	49 (36.0)	62 (33.5)		42 (32.8)		20 (35.1)	
**Demographic Characteristics**							
**Age in years**			0.237^a^		0.593^a^		0.060^a^
<30	58 (40.6)	75 (38.5)		50 (37.3)		25 (41.0)	
30–40	44 (30.8)	76 (39.0)		49 (36.6)		27 (44.3)	
>40	41 (28.7)	44 (22.6)		35 (26.1)		9 (14.8)	
Mean±SD	33.8±9.5	33.2±10.4	0.589^b^	34.4±11.0	0.665^b^	30.8±8.3	**0.029** ^**b**^
**Gender**			0.526^a^		0.672^a^		0.466^a^
Male	52 (35.6)	76 (39.0)		51 (38.1)		25 (41.0)	
Female	94 (64.4)	119 (61.0)		83 (61.9)		36 (59.0)	
**Ethnicity**			**0.016** ^**a**^		**0.001** ^**a**^		0.428^a^
Bedouins	63 (43.2)	55 (28.2)		31 (23.1)		24 (39.3)	
Non-Bedouins	64 (43.8)	106 (54.4)		74 (55.2)		32 (52.5)	
Persians	19 (13.0)	34 (17.4)		29 (21.6)		5 (8.2)	
**Migration history during Gulf War (1990/91)**			**0.017** ^**a**^		**0.008** ^**a**^		0.364^a^
Full domicile in Kuwait	71 (53.0)	127 (66.1)		91 (68.9)		36 (60.0)	
Migrated outside Kuwait	63 (47.0)	65 (33.9)		41 (31.1)		24 (33.8)	
**Sociocultural Characteristics**							
**Smoking history**			**0.026** ^**a**^		0.113^a^		**0.012** ^**a**^
Negative	118 (83.1)	142 (72.8)		101 (75.4)		41 (67.2)	
Positive	24 (16.9)	53 (27.2)		33 (24.6)		20 (33.8)	
**Family history of MS**			**<0.001** ^**a**^		**<0.001** ^**a**^		**<0.001** ^**a**^
Negative	133 (95.7)	144 (73.8)		95 (70.9)		49 (80.3)	
Positive	6 (4.3)	51 (26.2)		39 (29.1)		12 (19.7)	
**History of chronic comorbidity**			**0.005** ^**a**^		**0.001** ^**a**^		0.645^a^
No	101 (73.2)	110 (58.2)		68 (52.7)		42 (70.0)	
Yes	37 (26.8)	79 (41.8)		61 (47.3)		18 (30.0)	
**Daily sunlight exposure (min/day)**			**<0.001** ^**a**^		**<0.001** ^**a**^		**0.001** ^**a**^
<15	68 (49.3)	155 (79.5)		109 (81.3)		46 (75.4)	
≥15	70 (50.7)	40 (20.5)		25 (18.7)		15 (24.6)	
**Mode of routine outdoor dressing**			0.654^a^		0.980^a^		**0.026** ^**a**^
Partially shrouded dressing	112 (83.0)	158 (81.0)		116 (86.6)		42 (68.9)	
Fully shrouded dressing	23 (17.0)	37 (19.0)		18 (13.4)		19 (31.1)	

Numbers may not add up to the total due to missing values.

Significant p values are presented in bold font.

*Controls vs All MS patients;

^†^Controls vs Established MS Patients;

^‡^Controls vs Newly diagnosed patients.

p values are generated by

^a^chi-square test,

^b^Student t-test,

^c^Fishers exact test and

^d^Mann-Whitney U-test.

The results obtained, as indicated on Table 3, clearly demonstrate that:

**Table 3:** Environmental and sociocultural factors associated with multiple sclerosis in Kuwait selected by multiple logistic regression analysis.

	All patients		Established MS patients		Newly diagnosed patients (drugs naïve)	
Variables	AOR* (95% CI)	p	AOR (95% CI)	p	AOR (95% CI)	p
**Smoking status**						
Never	1.0				1.0	
Ever	1.9 (0.8–4.5)	0.128	1. 1 (0.4–2.9)	0.880	5. 2 (1.4–18.5)	**0.012**
**Ethnicity**						
Bedouins	1.0		1.0		1.0	
Non-Bedouins	2 (1.0–3.9)	**0.049**	2.0 (0.9–4.6)	0.088	2 (0.8–4.8)	0.143
Persians	1.47 (0.6–3.6)	0.399	2 (0.7–5.3)	0.178	0.6 (0.2–2.6)	0.514
**Family history of MS**						
Negative	1.0		1.0		1.00	
Positive	10.6 (3.0–36.9)	**<0.001**	16.1 (4.2–61.9)	**<0.001**	5.3 (1.3–21.8)	**0.021**
**History of chronic comorbidity**						
No	1.0		1.0		1.0	
Yes	2.4 (1.3–4.7)	**<0.001**	4 (1.8–8.4)	**0.001**	1. 4 (0.6–3.3)	0.497
**Sunlight exposure (min/day)**						
≥15	1.0		1.0		1.0	
<15	5.3 (2.7–10.5)	**<0.001**	5.6 (2.6–12.2)	**<0.001**	7.5 (2.6–21.4)	**<0.001**
**Mode of routine outdoor dressing**						
Partially shrouded dressing	1.0		1.0		1.0	
Fully shrouded dressing	2.2 (1–5)	0.065	1.1 (0.4–3.2)	0.824	4.0 (1.5–10.7)	**<0.001**

*AOR = Adjusted Odds Ratio. Included variables in the model are: age, sex, BMI, Kuwaiti Arabs, smoking status, migration history during Gulf War (1990/91), family history of MS, history of associated diseases, sunlight exposure (min/day); mode of routine outdoor dressing.

Significant p values are presented in bold font.

1. A positive family history of MS was associated with a higher risk of MS. Fifty one (26.2%) patients had a family history of MS—this was first degree in 46% (clinically definite MS was documented in 19 siblings—14 females, 5 males). Furthermore, consanguinity was reported in 42 patients (22.5%). The corresponding odds ratio of MS in those reporting a family history of MS was therefore high [AOR 10.6, 95% CI: 3.0–36.9, p <0.001)].
2. Duration of daily sunlight exposure was found related to prevalence of MS. While 50.7% (n = 70) controls reported ≥15 min/day sun exposure, only 20.5% (n = 40) of patients attained that degree of exposure (p<0.001) (AOR 5.3, 95% CI: 2.7–10.5). This finding persisted even after adjustment for age, sex, BMI, smoking, clothing preferences, family history of MS and history of comorbidities.
3. Ethnicity also appeared to increase MS risk. As earlier indicated, there is a degree of historical heterogeneity in the Kuwaiti Arab population which has translated to tangible differences in social and cultural norms that may be related to MS susceptibility. In this context, it was established that subjects classified as of Bedouin ethnicity appeared to be more protected than non-Bedouin Kuwait Arabs against MS occurrence (AOR 2, 95% CI: 1.00–3.9, p 0.049), even after adjustment for age, sex and BMI.
4. The patients with MS had a greater frequency of chronic comorbidities than the control subjects. Indeed, 110 (58.2%) of the MS patients reported no physical comorbidities, while 79 (41.8%) reported at least one. There is therefore a suggestion that presence of associated chronic medical illnesses was somewhat related to increased risk of MS (AOR 2.4, 95% CI: 1.3–4.7, p<0.001) (Table 3). The most frequent comorbidities encountered were: migraine (6.3%), osteoporosis (6.3%), hypothyroidism (5.8%), asthma (5.3%) and epilepsy (2.6%). These illnesses were not seen in the control population.
  We also made some observations on associations in the MS population that did not attain statistical significance in the whole group of subjects but appeared variably important in the sub-groups of patients (i.e. those with established disease or those that were drug-naïve with newly diagnosed disease). These observations were related to:
  1. Current (or past) smoking: Table 2 suggested smoking as more common in MS patients (27.2%) than in controls (16.9%) (p 0.026). However, after adjustment for gender and age, it was established that this significance persisted only in the newly diagnosed cohort of patients, with the suggestion that the newly diagnosed patients were at increased risk of MS compared with non-smokers (AOR 5.2, 95% CI: 1.4–18.5, p 0.012).
  2. Mode of outdoor clothing appeared to influence MS risk, but only in the newly diagnosed patients, in whom routine dressing in fully shrouded clothing was more common (31.1%) than in patients with established MS (13.4%) and controls (17%) (p 0.026, Table 2). Indeed, this observation remained significant in this group of patients only, even after adjustment for confounding (p < 0.001, Table 3). For the whole group, there was the sense of a potential relationship (p 0.065) after adjustment for confounding, with the suggestion that the association might have attained statistical significance with higher subject numbers.
5. Individuals who stayed in Kuwait throughout the 1990/91 Gulf War appeared more likely to develop MS than those who did not (p 0.017, Table 2). Indeed, about 66% of the MS patients and 53% of the controls spent their time in Kuwait during the war time period. However, this relationship was not confirmed on multivariate analyses.

## Discussion

There has been some debate on the changing prevalence of MS in Kuwait. Two recent hospital-based studies reported increased incidence in the last 2 decades [5–7]. The most recent study (7) suggested that Kuwait had emerged as a high risk zone as delineated by Kurtzke [10]. What is certainly clear is that more cases are currently being diagnosed as MS, but this may be related to better diagnostic ascertainment from improvement in specialist manpower and technology over the years. In addition, active MS support groups have, through educational programs, made the population more aware of the disease.

Nonetheless, it is important to attempt to unravel causes and associations of this distressing disease, from a population perspective, in order to advise on potential preventative measures.

To that extent, the results from this case-control study suggest that a positive family history of MS, certain associated medical illnesses (particularly epilepsy, migraine, hypothyroidism, seen in our group of patients), and possibly (p 0.065), routine outdoor dressing involving full body shrouding, appear to be associated with increased risk of MS. Protective factors may include relatively increased daily sunlight exposure and Bedouin ethnic extraction. These factors are considered in more detail.

We have reported an inverse association between sunlight exposure and MS risk in keeping with some previous reports [11–14]. Indeed, a recent Iranian study, in confirming our observations, also suggested that sunlight exposure of > 45 min/day could potentially translate into a 70% lower risk of MS [15]. The classical explanation for the link between sunlight exposure and MS risk is related to vitamin D status, although the specific mechanisms are yet unproven or even consistently demonstrable [16]. A study from Australia suggested that both solar radiation and vitamin D exert independent protective influences on MS development [14] by independently stimulating T-regulatory cells and secretion of IL-10, reducing levels of pro-inflammatory cytokines IL-17 and dampening T-helper (Th-1) immune function [14]. We find these hypotheses fascinating and have included measurement of vitamin D indices in our further studies, especially in a country like Kuwait with almost year round sunlight exposure, albeit with cultural practices that enhance sun avoidance. Indeed, as indicated above, women with newly diagnosed MS (but not those with established disease) who are fully shrouded except for their eyes and palms, as part of cultural and religious norms, appear to be at a higher risk of developing MS, compared to those wearing western dressing or those who, although essentially fully covered up, left their faces and hands exposed. These novel observations are worthy of further detailed study in an anthropological perspective.

Another potentially important observation from this study is that Kuwaiti Arabs of Bedouin ethnicity appeared to have a lower risk of developing MS (p 0.049), with the implication that those Kuwaiti Arabs of non-Bedouin and Persian extraction had a 1.5–2.0 times higher risk for MS, respectively, compared with those of Bedouin ethnicity. This finding would be in keeping with a previously report from Alter et.al. [17], that MS prevalence rates were relatively low among Arab Bedouins from the Negev desert in Israel. However, that study calculated a prevalence rate of 17.3 per 10^5^ in Negev Bedouins, which was essentially similar to the low MS prevalence rates for other Israeli Arab ethnicities [17]. The observation is even more intriguing, as Bedouin culture is considered conservative, with expectations of women’s full shrouding outdoors and consequent reduced sun exposure and perhaps a higher risk for MS.

We believe by virtue of the fact that our subjects and controls were randomly recruited and matched for age, gender, education level and region of residence, the possibility of selection bias was unlikely. There are many potential explanations for this ‘Bedouin paradox’. Despite westernization of Kuwaiti societies, Bedouins still maintain their cultural ties and religious practices [18] and retain isolated but close tribal communities, with large family sizes [18, 19] and possibly increased exposure to endemic infectious agents. Consistent with the “hygiene hypothesis” therefore, it could be proposed that early life exposure to common childhood infections in Bedouins, may have contributed to their lower risk of MS [2]. Additionally, genome-wide studies have shown that 17% of the Bedouins’ genome can be traced to Africans ancestry, with the possibility of inheritance of MS resistant genetic traits [4].

The importance of inherited genetic traits is reinforced by our observation that a positive family history of MS is a significant risk factor for the MS development. Indeed, in univariate analysis, it was established that 26.2% of MS patents reported a family history of MS as compared to 4.3% of healthy controls, (AOR 10. 6, 95% CI 3.0–36.9, p <0.001), an observation that is consistent with reports from previous Western studies [20,21]. The highest reported family aggregation of MS among cases from Arab populations was 9.3%, which is much lower than the rate reported in the present study [22]. We believe that our higher frequency of familial MS in comparison to other Arab populations is likely related to diversity in studies design and degree of case ascertainment, rather than to variable genetic predisposition. In breaking down the distribution of familial MS further, we demonstrated that, in those patients with positive family history of MS, 19 (37.3%) had at least one sibling with clinically definite MS. We were however unable to calculate the MS sibling recurrence risk in our subjects because we had no robust data on the total sibling counts of patients with positive family history.

Also of some interest is the fact that 42 (22.5%) patients were considered offspring’s of consanguineous marriages. This finding is consistent with the high rate of consanguinity in Arab populations [23]. A previous small study from Saudi Arabia had suggested that MS patients with a positive family history of MS were more likely to be offsprings of consanguineous marriages [24]. Our current study is in some agreement, in spite of limitations posed by likely under-reporting of family history because of cultural norms around truthfully disclosing family chronic illnesses to unrelated third parties. Nonetheless, we suggest that the relatively high rate of positive family history of MS we have identified is perhaps related to genetic factors and its association with consanguinity [23]. These hypotheses deserve further study.

This study has also established that the presence of certain comorbidities appeared to be associated with increased risk of MS. This observation should be considered soft because of the relatively small percentages of the total subject numbers with associated chronic morbidities. Indeed, while the respective frequencies of asthma, thyroid disease, epilepsy and osteoporosis in our patients are comparable to those reported from other studies, it would appear that other illnesses such as depression, migraine, hypertension and dyslipidemia were present in lesser frequencies in our MS population [25] These discrepancies are worthy of further study with much larger subject numbers, possibly in an international study context, as MS remains a relatively uncommon disease.

Another important factor evaluated in this study is the potential impact of the 1990/91 Gulf War and the environmental destruction consequent on burning oil wells. We could establish that individuals who remained in Kuwait throughout that period appeared more likely to develop MS (66.1%) as compared to those who did not (53%), (p 0.017). This significant association could not be replicated on multivariate analysis, probably because our subject numbers were relatively small. A more focused study might, in future, provide further clues on the likely environmental influences on MS pathogenesis.

We observed a positive association between smoking and MS only in our cohort of newly diagnosed, drugs naïve MS patients, whose risk of MS is 5 times higher compared with controls. This is worthy of some comment. Epidemiological studies have been inconclusive on the association between smoking and MS susceptibility. Some recent case-control [20,21,26,27] and cohort studies [28–31] support our findings while at least one other, from Italy, is in disagreement [32], perhaps as a result of differences in study design and methodology of ascertainment. Nonetheless, the biological basis of the putative link between smoking and MS risk is unclear. It is recognized that cigarette smoke affects several immune functions that are potentially important in the genesis of MS—these include T cell, B cell, and natural killer cell functions [33]. Furthermore, tobacco smoke contains nitric oxide and cyanide, both of which have been shown to cause axonal degeneration or to block axonal conduction, especially in demyelinated axons [34]. It is our intention to expand our studies to investigate these potential etiologic relationships in drug naïve MS subjects further.

This study has major strengths in its relatively large numbers for a small country, inclusion of incident MS cases, stringency of criteria used for establishing the diagnosis of definite MS and the availability of robust information on potential confounders such as family history and ethnicity in a relatively homogenous population. However, there are also limitations that should detract from an uncritical acceptance of the study conclusions. Although the patients and controls were matched in terms of their ethnicity, place of birth and residence, the number of the patients and controls were still relatively small as reflected by the large confidence intervals of the odd ratios. Furthermore, as in most case-control studies, where history of sunlight exposure and cigarette smoking (current and past) is obtained retrospectively, exposure misclassification may have occurred.

In conclusion, the study has demonstrated that a family history of MS and presence of certain co-morbidities appear to be associated with increased risk of MS. On the other hand, it was suggested that above-average outdoor sunlight exposure and Bedouin ethnicity were associated with a lower risk of MS. We have suggested a likely etiopathogenic role for vitamin D status, in view of the observations on sunlight exposure, and this will be the basis for our further studies in the same population. We believe that these observations provide a foundation for other anthropological and socio-demographic studies on other nutritional, environmental and socio-cultural determinants of MS disease risk in populations that appear to be experiencing increasing prevalence of the distressing disorder.
